# Autoimmune Psychosis Exists: Early Diagnosis of an Anti‐NMDA Receptor Autoimmune Encephalitis Presenting as First‐Episode Psychosis in a 25‐Year‐Old Male—A Case Report

**DOI:** 10.1155/crps/3931587

**Published:** 2025-12-30

**Authors:** Marc Mourad, Caroline Hallal, Juliana Sargi, Elie Atallah, Anthony Kassab, Sajida Sabsaby, Christian Matta, Karine Abou Khaled, Sami Richa

**Affiliations:** ^1^ Department of Psychiatry, Psychiatric Hospital of The Cross, Jal El Dib, Maten, Lebanon; ^2^ Department of Psychiatry, Faculty of Medicine, Saint Joseph University of Beirut, Beirut, Lebanon, usj.edu.lb; ^3^ Department of Psychiatry, University of Tours, Tours, France, univ-tours.fr; ^4^ Department of Psychology and Social Sciences/School of Medicine and Medical Sciences, Holy Spirit University of Kaslik, Jounieh, Lebanon, usek.edu.lb; ^5^ Department of Neurology, Faculty of Medicine, Saint Joseph University of Beirut, Beirut, Lebanon, usj.edu.lb

**Keywords:** autoimmune diseases, early diagnosis, encephalitis, extreme delta brush EEG aspect, first episode of psychosis, immunotherapies, psychosis

## Abstract

**Background:**

Anti‐N‐methyl‐D‐aspartate receptor (NMDAR) encephalitis is an autoimmune disorder marked by prominent neuropsychiatric symptoms. It is typically first encountered by psychiatrists because psychotic symptoms can be early signs of the condition. In recent years, this form of encephalitis has been established as a distinct diagnostic entity in neurology and psychiatry. Furthermore, as an organic and autoimmune psychosis, it is considered a differential diagnosis of schizophrenia and schizoaffective disorders.

**Clinical Presentation:**

We report the case of a 25‐year‐old male who presented with a first episode of psychosis. During his stay, the patient exhibited severe cognitive deficits (disorientation, confusion, memory issues), movement disorders (dysarthria, perioral dyskinesia leading to speech difficulties), a decreased level of consciousness, and a catatonic state complicated by a malignant neuroleptic syndrome. The patient also experienced epileptic seizures and had unstable vital signs. An electroencephalogram (EEG) revealed an extreme delta brush pattern (specific for anti‐NMDAR encephalitis), and CSF analysis showed an elevated immunoglobulin G (IgG) index. Based on these findings, anti‐NMDAR autoimmune encephalitis was suspected 17 days after admission but not yet confirmed. The patient was treated with oral corticosteroids followed by plasmapheresis and showed significant improvement. At discharge, he was alert, oriented, cooperative, and not psychotic, with only mild cognitive defects. Days after discharge, anti‐NMDAR IgG antibodies were detected in his CSF, confirming the diagnosis.

**Clinical Presentation:**

This case underscores the importance of considering anti‐NMDAR autoimmune encephalitis as a differential diagnosis in patients with no personal or family psychiatric history who develop subacute psychotic symptoms (lasting less than 3 months) along with fluctuating neuropsychiatric signs. Conducting an EEG, cerebral MRI, and CSF analysis to confirm or exclude the condition, followed by early immunosuppressive treatment, is crucial for improving prognosis.

## 1. Introduction

Since the exact pathophysiology of schizophrenia spectrum and other psychotic disorders is not yet fully understood, these disorders remain as theoretical constructs describing clinical syndromes rather than clearly defined diseases, especially given the lack of identifiable biomarkers [1]. These disorders are typically diagnosed after excluding medical and substance‐induced psychotic disorders [1].

For this reason, the Diagnostic and Statistical Manual of Mental Disorders, Fifth Edition, Text revision (DSM‐5‐TR) [2], and the International Statistical Classification Of Diseases and Related Health Problems, 11^th^ edition (ICD‐11) [3] maintained the exclusion of diagnoses related to alcohol, drugs, systemic issues, or other organic brain causes [1]. In fact, excluding these causes is a crucial step before diagnosing any schizophrenia spectrum and other psychotic disorders [1].

Neuroinflammatory diseases affecting the central nervous system (CNS), such as multiple sclerosis and vasculitis, or systemic autoimmune diseases like systemic lupus erythematosus (SLE), autoimmune thyroiditis, or the relatively new condition, anti‐N‐methyl‐D‐aspartate receptor (NMDAR) autoimmune encephalitis, described by Dalmau in 2007, are possible causes of “organic psychoses” [4]. These etiologies should be considered as differential diagnoses, especially when rapidly developing psychotic signs and symptoms (less than 3 months) are accompanied by fluctuating neuropsychiatric signs in a previously healthy individual without a family or personal psychiatric history [5]. Anti‐neuronal antibodies such as NMDAR, LGI1 (leucine‐rich glioma inactivated 1), Caspr2 (contactin‐associated protein 2), DPPX (dipeptidyl‐peptidase‐like protein 6), GABA (gamma‐aminobutyric acid), mGluR5 (metabotropic glutamate receptor 5) and GlyR (glycine receptor) target synapses and neuronal cell surface antigens and are linked to the mental symptoms of these autoimmune encephalitides [6].

In our clinical case, we describe a young male patient with anti‐NMDAR autoimmune encephalitis who presented with a first episode of psychosis, severe cognitive deficits, movement disorders, altered consciousness, catatonia, epilepsy, and unstable vitals. We highlight the red flags that should alert clinicians to the possible, probable, or definite diagnosis of anti‐NMDAR encephalitis, along with the first‐line treatments that improve prognosis through early intervention.

## 2. Case Presentation

A 25‐year‐old man arrived at the emergency department of our university hospital in Beirut due to agitation, disorganized behavior, confusion and dysarthria (Day 1). Previous non injected cerebral magnetic resonance imaging (cMRI) and electroencephalogram (EEG) tests, performed in an outpatient setting because the patient had experienced seizures later deemed nonepileptic, were normal. The patient had been prescribed up to 20 mg of escitalopram daily by an external psychiatrist with no improvement. The parents reported no history of drug misuse. The initial differential diagnosis at the emergency department was a “brief psychotic disorder”, and the patient was started on risperidone 2 mg daily, diazepam 15 mg daily, and chlorpromazine 100 mg daily. He was admitted to the psychiatric ward without further investigation other than a routine blood test, including CBC, which showed normal renal, hepatic, and thyroid functions. However, dyslipidemia, hyperuricemia and elevated ESR were also noted in the blood work.

During his stay (Days 1–6), the patient’s condition worsened on all levels: He continued to be agitated and disorganized, the dysarthria persisted, and a perioral dyskinesia, which affected his speech, developed. Additionally, the patient remained disoriented in time and place, as well as towards his family members. According to his family, memory problems had been present for months: he called them multiples times a day and asked to speak to his mother without remembering that he already done so before.

During his stay (Days 1–6), the patient also experienced auditory and visual hallucinations: He frequently talked to himself and appeared as if he was seeing someone. The risperidone dosage was increased to 8 mg per day without any response. The patient was also administered multiple antipsychotics in injectable (intramuscular) forms to help calm his agitation.

A first EEG on Day 7 was not clinically significant and showed artifacts without any epileptic discharges. creatine phosphokinase (CPK) levels reached 2459 U/L with normal kidney function after multiple antipsychotics injections. Hydration with 2 L of isotonic serum (physiological saline) was started daily.

From Day 8 to Day 12, the patient experienced a gradual loss of consciousness: His Glasgow Coma Scale (GCS) decreased even though he was not in a coma. Diazepam and chlorpromazine were immediately discontinued. CPK levels dropped to 1430 U/L on Day 8.

Over the next 2 days until Day 14, the patient progressively developed mutism, stupor, waxy flexibility, posturing, catalepsy, rigidity, mannerisms, stereotypes, and other signs of catatonia. Risperidone was immediately discontinued, and an electroconvulsive therapy (ECT) session was done after the anesthesia team’s permission to proceed: no clinical improvement was observed.

A second EEG was performed on Day 15 after the development of catatonia, revealing moderate diffuse cerebral dysfunction characterized by generalized slowing and attenuation of the basal rhythm with theta and delta waves. No epileptic seizures were recorded. An injected cerebral CT scan was also conducted and showed no abnormalities; a cMRI was not performed due to a technical issue at the hospital.

During the same night and for the first time during this hospital stay, the patient experienced an epileptic seizure: tonic‐clonic convulsions that lasted for 10 min. No medications were given. The next day (Day 16), he was diagnosed with pneumonia on a pulmonary CT scan, and antibiotics were started. He remained in the psychiatric ward despite desaturation and hypertension. ECT sessions were then discontinued.

On the same day (Day 16), the neurology team decided to proceed with a lumbar puncture (CSF analysis) after many neurologic and vegetative signs appeared during the hospital stay (cognitive deficits, movement disorders, loss of consciousness, catatonia, seizures and autonomic instability). Anti‐NMDAR autoimmune encephalitis was at this stage considered a possible diagnosis. In fact, we found out after a thorough interview with the parents that many neurological signs had also been present before hospitalization. The CSF analysis showed an increased immunoglobulin G (IgG) index: no infected CSF and no malignant cells on pathology. Research into anti‐NMDA IgG antibodies (Anti GluN1) and oligoclonal bands in the CSF was initiated in order to confirm the diagnosis of anti‐NMDAR autoimmune encephalitis.

A third EEG was performed on Day 17 and revealed an “extreme delta brush aspect: diffuse delta slowing of 1 3 Hz with superimposed bursts of rhythmic 20–30 Hz beta frequency activity “riding” on each delta wave.” This pattern was considered compatible with an anti‐NMDAR encephalitis, as it correlated with moderate diffuse cerebral dysfunction.

On the same day (Day 17), the patient was started on corticosteroids (intravenous methylprednisolone [IV], 1 g/24 h for 5 days: Day 17–Day 21) and anticonvulsants (valproic acid: 500 mg/12 h IV) because the combination of signs, symptoms, EEGs, and CSF findings pointed to a probable autoimmune encephalitis. The team did not wait for a definite diagnosis before beginning treatment. A testicular ultrasound was normal, ruling out paraneoplastic encephalopathy secondary to a testicular tumor. Other laboratory tests, including a total body scan, anti‐thyroid peroxidase (Anti‐TPO), and others, were not performed at the hospital due to financial constraints.

The patient became immediately more alert after the first dose of IV corticosteroids (Day 17): He responded to external stimuli but remained partially disoriented and dysarthric. A second generalized tonic–clonic seizure occurred on Day 18, during the second day of corticosteroids; no antiepileptics were administered, and the seizure stopped after 20 min. The patient’s vital signs improved over the 5 days of IV corticosteroids.

He was transferred to neurology on Day 20.

A fourth EEG on Day 22, 1 day after stopping the IV corticoids, showed that the “extreme delta brush aspect” was still present, although less pronounced.

On Day 23, 2 days after stopping the IV corticosteroids, the patient began receiving plasmapheresis (since intravenous immunoglobulin (IVIG) was unavailable). This decision was based on the lack of complete response to corticosteroids: Epilepsy persisted, and the patient exhibited echolalia and perseveration, suggesting a potential frontal lobe motor syndrome [7]. Five plasmapheresis sessions were carried out from Day 23 to Day 32.

During the sessions, several clinical incidents occurred: Notably, carbamazepine (200 mg/12 h) was added to valproic acid on Day 24 when the patient exhibited muscle twitches that were considered signs of epileptic seizures. The response to plasmapheresis began to emerge after the third session (Day 25): There was no longer perseveration, orientation improved, confusion decreased, the patient became more cooperative, and started responding to complex commands.

On Day 29, 4 weeks after the patient’s hospital admission, a video EEG was performed because the level of consciousness continued to fluctuate; it revealed epileptic seizures. Carbamazepine dosage was increased to 400 mg every 12 h, with clonazepam added as needed. A partial response was observed. On Day 33, levetiracetam was added to the treatment with valproic acid and carbamazepine since the patient continued presenting epileptic seizures: a 3000 mg IV loading dose, followed by 1500 mg every 12 h IV. These seizures persisted for 1 day after plasmapheresis ended.

On Day 35, the patient was transferred to the intensive care unit after developing a nosocomial pneumonia, as shown on a pulmonary CT scan, along with a high CRP and desaturation. He was intubated, then successfully extubated without complications, and left the ICU on Day 43 after receiving multiple antibiotics (vancomycin, levofloxacin, and tazocillin) with corticosteroids. He began physical therapy upon being transferred back to the neurology floor, once neurological and respiratory stability had been achieved. His neurological exam revealed an alert, well‐oriented, cooperative patient without dysarthria or perseveration. His pupils were symmetric and reactive. The motor strength was 4/5, and sensation was intact.

**Figure 1:**
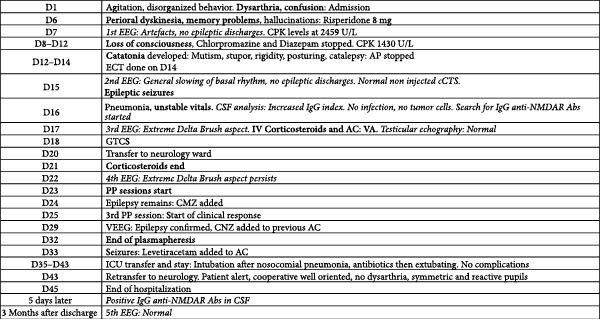
Clinical signs, diagnostic tools, and treatment strategies timeline. Bold: Clinical signs that helped the medical team suspect anti‐NMDA receptor autoimmune encephalitis as a possible diagnosis AND the treatments procedures that were used.Italics: The EEG and lumbar punction results. AC, anticonvulsants; AP, antipsychotics; cCTS, cerebral CT scanner; CNZ, clonazepam; CPK, creatine phosphokinase; CSF, cerebrospinal fluid; D, day; ECT, electroconvulsive therapy; EEG, electroencephalogram; GTCS, generalized tonic–clonic seizures; ICU, intensive care unit; IgG, immunoglobulin G; IV, intravenous; NMDAR Abs, N‐methyl‐D‐aspartate receptor antibodies; PP, plasmapheresis; VA, valproic acid; VEEG, video EEG.

The patient left the hospital on Day 45 while taking valproic acid (2500 mg daily), carbamazepine (1000 mg daily), levetiracetam (3000 mg daily), and clonazepam 0.5 mg as needed. Corticosteroids were also prescribed, specifically 50 mg of prednisone to be tapered over 2 weeks. The glutamate receptor IgG Abs (type NMDA/CSF) titer was positive (value: 1 : 128, standard range: <1 : 1 titer) 5 days after discharge and anti‐ NMDAR encephalitis was then confirmed as a definite diagnosis. Additional tests concluded outside the hospital, including HIV serology, hepatitis B and C, tuberculosis screening via interferon‐gamma release assay, thyroid antibody tests, and a PET scan, showed no abnormalities. Rituximab was considered as a treatment option in case of relapse. An EEG performed 3 months after discharge was normal. The patient’s recovery progressed well, though he experienced mild memory deficits and developed a depressive episode, which was treated with an SSRI. Figure 1 illustrates all the diagnostic and therapeutic steps.

## 3. Discussion

In our clinical case describing a patient presenting with rapidly developing psychotic and neurological signs, we observe that 16 days after admission, anti‐NMDAR encephalitis was suspected as a possible diagnosis, and a CSF analysis was performed to detect CNS autoimmune disturbances and search for antibodies. In addition, by 17 days post‐admission, when the CSF analysis showed an increased IgG index, and the EEG an extreme delta brush aspect, anti‐NMDAR autoimmune encephalitis was considered a probable diagnosis and the treatment was initiated. This time interval is relatively short in comparison to the literature. For example, a mean delay of 74 days between symptoms onset and antibody testing at the Charité Center for Autoimmune Encephalitis in Berlin, based on a sample of 100 patients with various types of encephalitides from 2013 to 2016 as reported by Herken and Prüss [5]. The suspicion of this encephalitis was based on a wide range of clinical signs and additional test findings in a young patient with no prior personal or family history of mental disorders who experienced a rapid onset of psychotic symptoms (less than 3 months) [8].

An international consensus on the approach to diagnosing and managing psychosis of suspected autoimmune origin was proposed and described by Pollak et al. in his position paper of 2020 published in *The Lancet* [6]. These proposed diagnostic criteria help distinguishing between possible, probable and definite autoimmune psychosis [6]. Practically, when subacute psychosis is accompanied by at least one of these seven signs and symptoms considered “red flags” (severe cognitive deficits, movement and speech disorders, deterioration of consciousness, catatonia and neuroleptic malignant syndrome (NMS), epileptic seizures, autonomic dysfunction or current/recent diagnosis of tumor), anti‐NMDAR autoimmune encephalitis should be suspected as a possible diagnosis. The following steps would include performing a cerebral MRI, an EEG, and a CSF analysis [1, 5, 6]. Suppose the EEG shows rhythmic slowing or extreme delta brush, which are encephalopathic changes, AND the CSF has an increased IgG index or oligo clonal bands. In that case, the NMDAR autoimmune encephalitis is a probable diagnosis [6]. The findings in our reported case included an extreme delta brush pattern and increased IgG index. Other findings that would make the diagnosis probable include bilateral brain abnormalities on T2‐weighted FLAIR MRI highly restricted to the temporal lobes OR pleocytosis of > 5 white blood cells per μL in the CSF [6]. The next step would be to check for IgG‐class anti‐neuronal antibodies in the CSF: If IgG GluN 1 antibodies (anti‐NMDAR) are detected, then NMDAR autoimmune encephalitis is a definite diagnosis [6]. Starting immunosuppression as soon as possible is vital (corticosteroids therapy, IVIG, or plasmapheresis to remove pathogenic antibodies are the preferred treatments (first‐line options) for patients with definite autoimmune encephalitis) [1]. Rituximab and other agents such as methotrexate, mycophenolate mofetil or cyclophosphamide are reserved for refractory cases [1]. Beginning treatment when the diagnosis is probable and not definite yet is possible and clinically justified to enhance prognosis [6]. In fact, early treatment protects also against relapses: Ciano‐Peterson et al. argued in their 2025 publication about predictive features of relapse in “*Neurology, Neuroimmunology and Neuroinflammation*” that early administration of first and second line immunotherapies could protect against further relapses of anti‐NMDAR encephalitis [9]. New treatment modalities other than those used actually are being searched: A preclinical rational for exploring approaches to modulate and manipulate parvalbumine neuronal function in order to treat anti‐NMDAR encephalitis was discussed by Feng et al. [10] in 2025 in the journal “*Brain*”. In fact, parvalbumine neurons are interneurons in the medical prefrontal cortex that are involved in functions like cognition and addiction, they maintain the excitatory‐inhibitory balance in the frontal lobe and participate in forming cortical neural oscillations [10]. When targeted by the IgG anti‐NMDAR antibodies, they sustain damage resulting in synaptic dysfunction and cortical excitation disruption [10]. Another essential point to mention is that we described anti‐NMDAR autoimmune encephalitis in a male patient, even though it is known to be a female‐predominant disease: Women account for 80% of cases according to Amugoda et al. [11] and the female/male ratio is 4/1 according to Steiner et al. [1]. It is typically linked to ovarian tertatoma in women, as reported by Câmara‐Pestana et al. [12] and Aoki et al. [13 ] who described anti‐NMDAR autoimmune encephalitis in young women presenting with subacute psychosis and neurological signs that did not respond to standard antipsychotic treatment. In both instances, an ovarian teratoma was identified through radiology. In 2020, Forrester et al. [14] described 4 cases of anti NMDAR encephalitis in women who had subacute installation of neuropsychiatric signs and did not respond to standard treatment procedures by antipsychotics: two of these patients had ovarian teratoma In fact, since 2011, anti‐NMDAR encephalitis is not considered as an exclusively paraneoplastic syndrome associated with ovarian teratoma but as the first identified autoimmune synaptic encephalitis that can also touches women without ovarian teratoma and men [15].

One limitation in this case maybe the absence of a cerebral MRI in our assessments, but according to literature, MRIs are unremarkable in about 50% of patients with autoimmune encephalitis, contrary to abnormalities found in 90% of EEGs and CSF diagnostics. In fact, a functional impairment of the brain can be detected by an EEG and not by an MRI, especially if no visible brain structural change have developed yet [1]. A 2025 study by Moreno‐Avellan et al. [16]demonstrated that EEG abnormalities, especially diffuse slowing and the extreme delta brush pattern, offer important diagnostic clues in patients suspected of anti‐NMDAR encephalitis. In fact, the extreme delta brush aspect is highly specific for NMDAR encephalitis, resulting in few false‐positive results. In our case, this EEG feature was observed on the third EEG performed on Day 17 [16].

In conclusion, this case report highlights the importance of considering organic etiologies such as autoimmune encephalitis (including NMDAR and nonNMDAR) as possible differential diagnoses when a patient presents with a subacute onset of pleomorphic neuropsychiatric signs and symptoms and has no prior personal or family history of mental disorders. Early suspicion of organic causes, especially in young adults presenting to a psychiatric emergency room, is crucial for an timely intervention and improved outcomes [17].

## Ethics Statement

This case report was conducted in accordance with ethical guidelines. Written informed consent to participate in the report was obtained from the patient. The study was approved by the HDF Ethics Committee under the code CEHDF2218 (November 2023).

## Conflicts of Interest

The authors declare no conflicts of interest.

## Funding

The authors received no specific funding for this work.

## Data Availability

The data that support the findings of this study are available from the corresponding author upon reasonable request.
